# Crystal structure of *catena*-poly[[di­aqua­cobalt(II)]-bis­[μ-5-(4-carb­oxy­ylato­phenyl)picolinato]-κ^3^ *N*,*O* ^2^:*O* ^5^;κ^3^ *O* ^5^:*N*,*O* ^2^-[di­aqua­cobalt(II)]-μ-1-[4-(1*H*-imidazol-1-yl)phen­yl]-1*H*-imidazole-κ^2^ *N* ^3^:*N* ^3′^]

**DOI:** 10.1107/S2056989015012190

**Published:** 2015-06-30

**Authors:** Guo-Wang Xu, Ye-Nan Wang, Hai-Bing Wang, Zhong-Long Wang

**Affiliations:** a College of Science, China Three Gorges University, Yichang 443002, People’s Republic of China; b College of Mechanical and Power Engineering, China Three Gorges University, Yichang 443002, People’s Republic of China

**Keywords:** crystal structure, 5-(4-carb­oxy­phen­yl)picolinate, 1-[4-(1*H*-imidazol-1-yl)phen­yl]-1*H*-imidazole, one-dimensional coordination polymer, cobalt(II) complex

## Abstract

The asymmetric unit of the title polymeric Co^II^ complex, [Co_2_(C_13_H_7_NO_4_)_2_(C_12_H_10_N_4_)(H_2_O)_4_]_*n*_, contains a Co^II^ cation, a 5-(4-carboxyl­atophen­yl)picolinate dianion, two coordination water mol­ecules and half of 1-[4-(1*H*-imidazol-1-yl)phen­yl]-1*H*-imidazole ligand. The Co^II^ cation is coordinated by two picolinate dianions, two water mol­ecules and one 1-[4-(1*H*-imidazol-1-yl)phen­yl]-1*H*-imidazole mol­ecule in a distorted N_2_O_4_ octa­hedral coordination geometry. The two picolinate dianions are related by an inversion centre and link two Co^II^ cations, forming a binuclear unit, which is further bridged by the imidazole mol­ecules, located about an inversion centre, into the polymeric chain propagating along the [-1-11] direction. In the crystal, the three-dimensional supra­molecular architecture is constructed by O—H⋯O hydrogen bonds between the coordinating water mol­ecules and the non-coordinating carboxyl­ate O atoms of adjacent polymeric chains.

## Related literature   

For the structure of a related 5-(4-carb­oxy­phen­yl)picolinate complex, see: Meng *et al.* (2012 ▸). For a related 1,4-bis­(1-imidazoly)benzene compound, see: Li *et al.* (2009 ▸).
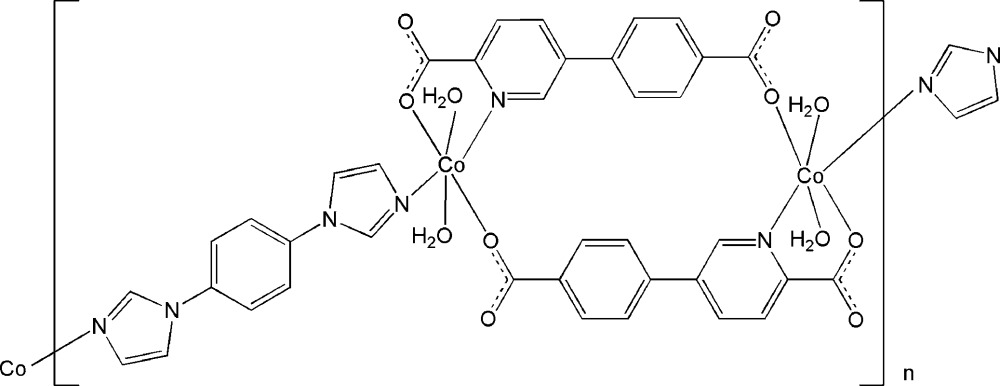


## Experimental   

### Crystal data  

[Co_2_(C_13_H_7_NO_4_)_2_(C_12_H_10_N_4_)(H_2_O)_4_]
*M* *_r_* = 441.28
Triclinic, 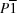

*a* = 7.055 (3) Å
*b* = 7.190 (3) Å
*c* = 20.038 (10) Å
α = 80.25 (2)°
β = 79.90 (2)°
γ = 64.080 (15)°
*V* = 895.1 (7) Å^3^
*Z* = 2
Mo *K*α radiation
μ = 1.00 mm^−1^
*T* = 293 K
0.20 × 0.20 × 0.17 mm

### Data collection  

Bruker SMART 1000 CCD diffractometer
Absorption correction: multi-scan (*SADABS*; Bruker, 2001 ▸) *T*_min_ = 0.825, *T*_max_ = 0.848
9552 measured reflections
4104 independent reflections
3636 reflections with *I* > 2σ(*I*)
*R*_int_ = 0.045

### Refinement  

*R*[*F* ^2^ > 2σ(*F* ^2^)] = 0.038
*wR*(*F* ^2^) = 0.090
*S* = 1.05
4104 reflections
274 parameters
4 restraints
H atoms treated by a mixture of independent and constrained refinement
Δρ_max_ = 0.34 e Å^−3^
Δρ_min_ = −0.41 e Å^−3^

### 

Data collection: *SMART* (Bruker, 2007 ▸); cell refinement: *SAINT* (Bruker, 2007 ▸); data reduction: *SAINT*; program(s) used to solve structure: *SHELXS97* (Sheldrick, 2008 ▸); program(s) used to refine structure: *SHELXL97* (Sheldrick, 2008 ▸); molecular graphics: *SHELXTL* (Sheldrick, 2008 ▸); software used to prepare material for publication: *publCIF* (Westrip, 2010 ▸).

## Supplementary Material

Crystal structure: contains datablock(s) I, New_Global_Publ_Block. DOI: 10.1107/S2056989015012190/xu5853sup1.cif

**Fig. 1.**
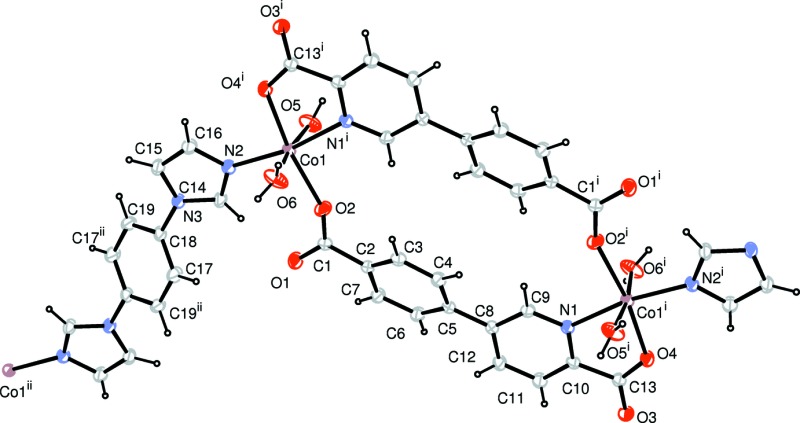
A part of the crystal structure of the title compound with labelling and displacement ellipsoids drawn at the 30% probability level. Symmetry codes: (i) -x, 1 - y, 1 - z; (ii) 1 - x, 2 - y, -z.

Structure factors: contains datablock(s) I. DOI: 10.1107/S2056989015012190/xu5853Isup2.hkl

x z x y z . DOI: 10.1107/S2056989015012190/xu5853fig1.tif
A part of the crystal structure of the title compound with labelling and displacement ellipsoids drawn at the 30% probability level. Symmetry codes: (i) −*x*, 1 − y, 1 − *z*; (ii) 1 − *x*, 2 − *y*, −*z*.

CCDC reference: 1062328

Additional supporting information:  crystallographic information; 3D view; checkCIF report

## Figures and Tables

**Table 1:** Selected bond lengths ()

Co1N1^i^	2.1403(18)
Co1N2	2.0815(18)
Co1O2	2.1575(16)
Co1O4^i^	2.1028(16)
Co1O5	2.0773(18)
Co1O6	2.0889(19)

Symmetry code: (i) 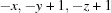.

**Table 2:** Hydrogen-bond geometry (, )

*D*H*A*	*D*H	H*A*	*D* *A*	*D*H*A*
O5H5*A*O3^ii^	0.81(2)	1.89(2)	2.697(3)	178(4)
O5H5*B*O1^iii^	0.82(2)	2.01(2)	2.825(4)	175(3)
O6H6*A*O3^iv^	0.81(3)	1.98(3)	2.769(3)	165(3)
O6H6*B*O1^v^	0.81(2)	1.99(2)	2.795(3)	171(3)

Symmetry codes: (ii) 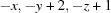; (iii) 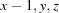; (iv) 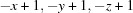; (v) 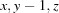.

## supplementary crystallographic information

### S1. Comment

Recent years, many successful examples have been made based on mixed ligands. Pyridine carboxylic acids and N-donor ligand as good kinds of organic linkers were always selected to construct various metal-organic frameworks (MOFs) with specially properties. In this work, we choose 5-(4-carboxyphenyl)picolinic acid and 1,4-bis(1-imidazoly)benzene to construct a new kind of Co-based MOFs. By now, a related 5-(4-carboxyphenyl)picolinate complex (Meng et al., 2012) and a related 1,4-bis(1-imidazoly)benzene compound (Li et al., 2009) have been reported.

### S2. Experimental

An aqueous mixture of cobalt(II) nitrate hexahydrate (58.2 mg, 0.2 mmol), 5-(4-carboxyphenyl)picolinic acid 48.6 mg, 0.2 mmol) and 1,4-bis(1-imidazoly)benzene (42 mg, 0.2 mmol) was placed in a Teflon-lined, stainless-steel reactor. The reactor was heated to 413 K for 72 hours. It was then cooled to room temperature at the rate of 15 K per hour. Red crystals were isolated in 73% yield (based on Co). C, H, N elemental analysis. calcd.for C19 H16 O6 N3 Co: C 51.71, H 3.65, N 9.52%; found C 51.88, H 3.71,N 9.92%.

### S3. Refinement

Water H atoms were located in a difference Fourier map and refined with a distance constraint of O—H = 0.82 (1) Å, U_iso_(H) = 1.5U_eq_(O). Other H atoms were positioned geometrically and refined using a riding model with C–H = 0.93 Å, U_iso_(H) = 1.2U_eq_(C).

### Crystal data

**Table:**

[Co_2_(C_13_H_7_NO_4_)_2_(C_12_H_10_N_4_)(H_2_O)_4_]	*Z* = 2
*M**_r_* = 441.28	*F*(000) = 452
Triclinic, *P*1	*D*_x_ = 1.637 Mg m^−^^3^
Hall symbol: -P 1	Mo *K*α radiation, λ = 0.71073 Å
*a* = 7.055 (3) Å	Cell parameters from 2498 reflections
*b* = 7.190 (3) Å	θ = 2.1–27.6°
*c* = 20.038 (10) Å	µ = 1.00 mm^−^^1^
α = 80.25 (2)°	*T* = 293 K
β = 79.90 (2)°	Prism, red
γ = 64.080 (15)°	0.20 × 0.20 × 0.17 mm
*V* = 895.1 (7) Å^3^	

### Data collection

**Table:**

Bruker SMART 1000 CCD diffractometer	4104 independent reflections
Radiation source: fine-focus sealed tube	3636 reflections with *I* > 2σ(*I*)
Graphite monochromator	*R*_int_ = 0.045
phi and ω scans	θ_max_ = 27.6°, θ_min_ = 3.1°
Absorption correction: multi-scan (*SADABS*; Bruker, 2001)	*h* = −9→9
*T*_min_ = 0.825, *T*_max_ = 0.848	*k* = −9→9
9552 measured reflections	*l* = −26→25

### Refinement

**Table:**

Refinement on *F*^2^	Primary atom site location: structure-invariant direct methods
Least-squares matrix: full	Secondary atom site location: difference Fourier map
*R*[*F*^2^ > 2σ(*F*^2^)] = 0.038	Hydrogen site location: inferred from neighbouring sites
*wR*(*F*^2^) = 0.090	H atoms treated by a mixture of independent and constrained refinement
*S* = 1.05	*w* = 1/[σ^2^(*F*_o_^2^) + (0.035*P*)^2^ + 0.341*P*] where *P* = (*F*_o_^2^ + 2*F*_c_^2^)/3
4104 reflections	(Δ/σ)_max_ = 0.001
274 parameters	Δρ_max_ = 0.34 e Å^−^^3^
4 restraints	Δρ_min_ = −0.41 e Å^−^^3^

### Special details

**Table:**

Geometry. All esds (except the esd in the dihedral angle between two l.s. planes) are estimated using the full covariance matrix. The cell esds are taken into account individually in the estimation of esds in distances, angles and torsion angles; correlations between esds in cell parameters are only used when they are defined by crystal symmetry. An approximate (isotropic) treatment of cell esds is used for estimating esds involving l.s. planes.
Refinement. Refinement of *F*^2^ against ALL reflections. The weighted *R*-factor *wR* and goodness of fit *S* are based on *F*^2^, conventional *R*-factors *R* are based on *F*, with *F* set to zero for negative *F*^2^. The threshold expression of *F*^2^ > σ(*F*^2^) is used only for calculating *R*-factors(gt) *etc*. and is not relevant to the choice of reflections for refinement. *R*-factors based on *F*^2^ are statistically about twice as large as those based on *F*, and *R*- factors based on ALL data will be even larger.

### Fractional atomic coordinates and isotropic or equivalent isotropic displacement parameters (Å^2^)

**Table:**

	*x*	*y*	*z*	*U*_iso_*/*U*_eq_	
Co1	−0.00343 (4)	0.48246 (4)	0.218107 (13)	0.02287 (10)	
N1	0.1553 (3)	0.6510 (3)	0.69865 (8)	0.0240 (4)	
N2	0.1229 (3)	0.5882 (3)	0.12579 (9)	0.0286 (4)	
N3	0.2772 (3)	0.7531 (3)	0.04950 (8)	0.0291 (4)	
O1	0.2958 (3)	0.7975 (3)	0.25034 (8)	0.0359 (4)	
O2	0.1274 (3)	0.5901 (3)	0.28498 (8)	0.0368 (4)	
O3	0.3160 (3)	0.8566 (2)	0.81611 (8)	0.0325 (3)	
O4	0.1426 (3)	0.6548 (3)	0.83101 (7)	0.0341 (4)	
O5	−0.2808 (3)	0.7538 (3)	0.22533 (10)	0.0404 (4)	
O6	0.2678 (3)	0.2026 (3)	0.22547 (11)	0.0443 (4)	
C1	0.2182 (3)	0.6971 (3)	0.29511 (10)	0.0247 (4)	
C2	0.2347 (3)	0.7058 (3)	0.36903 (10)	0.0242 (4)	
C3	0.2170 (3)	0.5541 (3)	0.41956 (11)	0.0279 (4)	
H3	0.1991	0.4436	0.4078	0.033*	
C4	0.2260 (3)	0.5653 (3)	0.48755 (11)	0.0286 (4)	
H4	0.2190	0.4596	0.5204	0.034*	
C5	0.2452 (3)	0.7331 (3)	0.50710 (10)	0.0248 (4)	
C6	0.2625 (4)	0.8848 (3)	0.45621 (11)	0.0294 (5)	
H6	0.2748	0.9983	0.4681	0.035*	
C7	0.2618 (3)	0.8700 (3)	0.38789 (11)	0.0287 (4)	
H7	0.2794	0.9701	0.3546	0.034*	
C8	0.2451 (3)	0.7502 (3)	0.58043 (10)	0.0253 (4)	
C9	0.1620 (3)	0.6403 (3)	0.63224 (10)	0.0270 (4)	
H9	0.1085	0.5552	0.6198	0.032*	
C10	0.2369 (3)	0.7693 (3)	0.71770 (10)	0.0246 (4)	
C11	0.3202 (4)	0.8855 (4)	0.67012 (11)	0.0330 (5)	
H11	0.3740	0.9681	0.6840	0.040*	
C12	0.3220 (4)	0.8769 (4)	0.60140 (11)	0.0329 (5)	
H12	0.3752	0.9563	0.5690	0.039*	
C13	0.2319 (3)	0.7604 (3)	0.79457 (10)	0.0246 (4)	
C14	0.2106 (4)	0.7192 (4)	0.11613 (11)	0.0341 (5)	
H14	0.2254	0.7813	0.1508	0.041*	
C15	0.2261 (4)	0.6350 (4)	0.01419 (11)	0.0346 (5)	
H15	0.2507	0.6269	−0.0326	0.042*	
C16	0.1328 (4)	0.5334 (4)	0.06182 (11)	0.0332 (5)	
H16	0.0830	0.4409	0.0529	0.040*	
C17	0.5370 (4)	0.8784 (4)	0.06193 (11)	0.0373 (6)	
H17	0.5615	0.7960	0.1034	0.045*	
C18	0.3908 (4)	0.8791 (3)	0.02369 (10)	0.0286 (5)	
C19	0.3533 (4)	0.9994 (4)	−0.03836 (11)	0.0346 (5)	
H19	0.2557	0.9982	−0.0639	0.042*	
H5A	−0.288 (5)	0.869 (2)	0.2128 (14)	0.052*	
H5B	−0.403 (2)	0.767 (5)	0.2352 (15)	0.052*	
H6A	0.383 (3)	0.191 (5)	0.2064 (14)	0.052*	
H6B	0.263 (5)	0.090 (2)	0.2309 (15)	0.052*	

### Atomic displacement parameters (Å^2^)

**Table:**

	*U*^11^	*U*^22^	*U*^33^	*U*^12^	*U*^13^	*U*^23^
Co1	0.02701 (16)	0.02627 (15)	0.02110 (15)	−0.01781 (12)	−0.00122 (10)	−0.00029 (10)
N1	0.0276 (9)	0.0289 (9)	0.0216 (8)	−0.0184 (8)	−0.0011 (7)	−0.0018 (7)
N2	0.0365 (10)	0.0367 (10)	0.0220 (9)	−0.0261 (9)	−0.0009 (7)	0.0002 (7)
N3	0.0420 (11)	0.0399 (10)	0.0188 (8)	−0.0316 (9)	0.0007 (7)	−0.0013 (7)
O1	0.0512 (10)	0.0455 (9)	0.0246 (8)	−0.0345 (9)	−0.0010 (7)	−0.0021 (7)
O2	0.0505 (10)	0.0487 (10)	0.0291 (8)	−0.0366 (9)	−0.0077 (7)	−0.0026 (7)
O3	0.0429 (9)	0.0393 (9)	0.0288 (8)	−0.0294 (8)	−0.0038 (7)	−0.0044 (7)
O4	0.0496 (10)	0.0474 (9)	0.0220 (7)	−0.0378 (9)	−0.0002 (6)	−0.0017 (7)
O5	0.0300 (9)	0.0252 (8)	0.0647 (12)	−0.0139 (8)	−0.0014 (8)	0.0008 (8)
O6	0.0285 (9)	0.0289 (8)	0.0732 (13)	−0.0152 (8)	0.0035 (8)	0.0000 (9)
C1	0.0246 (10)	0.0273 (10)	0.0256 (10)	−0.0140 (9)	−0.0030 (8)	−0.0031 (8)
C2	0.0218 (10)	0.0308 (10)	0.0231 (10)	−0.0139 (9)	−0.0022 (7)	−0.0036 (8)
C3	0.0294 (11)	0.0306 (11)	0.0298 (11)	−0.0180 (9)	−0.0027 (8)	−0.0042 (9)
C4	0.0338 (12)	0.0310 (11)	0.0267 (11)	−0.0205 (10)	−0.0026 (8)	0.0007 (8)
C5	0.0228 (10)	0.0323 (10)	0.0235 (10)	−0.0158 (9)	−0.0010 (8)	−0.0031 (8)
C6	0.0390 (12)	0.0331 (11)	0.0270 (10)	−0.0250 (10)	−0.0037 (9)	−0.0035 (9)
C7	0.0374 (12)	0.0313 (11)	0.0240 (10)	−0.0217 (10)	−0.0047 (8)	0.0016 (8)
C8	0.0256 (10)	0.0323 (10)	0.0216 (10)	−0.0163 (9)	−0.0009 (8)	−0.0022 (8)
C9	0.0314 (11)	0.0331 (11)	0.0251 (10)	−0.0213 (9)	−0.0039 (8)	−0.0026 (8)
C10	0.0253 (10)	0.0283 (10)	0.0256 (10)	−0.0167 (9)	−0.0009 (8)	−0.0035 (8)
C11	0.0445 (13)	0.0420 (12)	0.0279 (11)	−0.0331 (11)	−0.0004 (9)	−0.0050 (9)
C12	0.0442 (14)	0.0445 (13)	0.0229 (10)	−0.0332 (12)	−0.0005 (9)	0.0007 (9)
C13	0.0271 (11)	0.0272 (10)	0.0236 (10)	−0.0156 (9)	−0.0018 (8)	−0.0028 (8)
C14	0.0519 (14)	0.0485 (13)	0.0187 (10)	−0.0381 (12)	0.0015 (9)	−0.0040 (9)
C15	0.0461 (14)	0.0486 (13)	0.0215 (10)	−0.0322 (12)	0.0015 (9)	−0.0066 (9)
C16	0.0468 (14)	0.0405 (12)	0.0271 (11)	−0.0323 (11)	−0.0012 (9)	−0.0055 (9)
C17	0.0531 (15)	0.0522 (14)	0.0211 (10)	−0.0385 (13)	−0.0090 (10)	0.0098 (10)
C18	0.0404 (13)	0.0369 (12)	0.0189 (9)	−0.0283 (10)	0.0020 (8)	−0.0014 (8)
C19	0.0433 (13)	0.0516 (14)	0.0233 (10)	−0.0339 (12)	−0.0081 (9)	0.0032 (9)

### Geometric parameters (Å, º)

**Table:**

Co1—N1^i^	2.1403 (18)	C3—H3	0.9300
Co1—N2	2.0815 (18)	C4—C5	1.396 (3)
Co1—O2	2.1575 (16)	C4—H4	0.9300
Co1—O4^i^	2.1028 (16)	C5—C6	1.393 (3)
Co1—O5	2.0773 (18)	C5—C8	1.495 (3)
Co1—O6	2.0889 (19)	C6—C7	1.392 (3)
N1—C9	1.338 (3)	C6—H6	0.9300
N1—C10	1.347 (2)	C7—H7	0.9300
N1—Co1^i^	2.1403 (18)	C8—C12	1.394 (3)
N2—C14	1.309 (3)	C8—C9	1.400 (3)
N2—C16	1.385 (3)	C9—H9	0.9300
N3—C14	1.354 (3)	C10—C11	1.384 (3)
N3—C15	1.383 (3)	C10—C13	1.526 (3)
N3—C18	1.435 (3)	C11—C12	1.386 (3)
O1—C1	1.257 (2)	C11—H11	0.9300
O2—C1	1.254 (2)	C12—H12	0.9300
O3—C13	1.251 (2)	C14—H14	0.9300
O4—C13	1.255 (2)	C15—C16	1.357 (3)
O4—Co1^i^	2.1028 (16)	C15—H15	0.9300
O5—H5A	0.810 (10)	C16—H16	0.9300
O5—H5B	0.813 (10)	C17—C19^ii^	1.386 (3)
O6—H6A	0.811 (10)	C17—C18	1.387 (3)
O6—H6B	0.814 (10)	C17—H17	0.9300
C1—C2	1.520 (3)	C18—C19	1.384 (3)
C2—C3	1.390 (3)	C19—C17^ii^	1.386 (3)
C2—C7	1.395 (3)	C19—H19	0.9300
C3—C4	1.392 (3)		
			
O5—Co1—N2	94.70 (8)	C6—C5—C8	121.13 (18)
O5—Co1—O6	172.08 (8)	C4—C5—C8	120.96 (18)
N2—Co1—O6	92.55 (8)	C7—C6—C5	121.32 (19)
O5—Co1—O4^i^	92.29 (8)	C7—C6—H6	119.3
N2—Co1—O4^i^	92.21 (7)	C5—C6—H6	119.3
O6—Co1—O4^i^	90.60 (8)	C6—C7—C2	120.39 (19)
O5—Co1—N1^i^	86.11 (8)	C6—C7—H7	119.8
N2—Co1—N1^i^	169.23 (7)	C2—C7—H7	119.8
O6—Co1—N1^i^	87.36 (8)	C12—C8—C9	116.25 (18)
O4^i^—Co1—N1^i^	77.02 (7)	C12—C8—C5	122.94 (18)
O5—Co1—O2	89.39 (8)	C9—C8—C5	120.80 (18)
N2—Co1—O2	97.93 (7)	N1—C9—C8	123.55 (18)
O6—Co1—O2	86.46 (8)	N1—C9—H9	118.2
O4^i^—Co1—O2	169.55 (6)	C8—C9—H9	118.2
N1^i^—Co1—O2	92.81 (7)	N1—C10—C11	121.47 (19)
C9—N1—C10	119.08 (17)	N1—C10—C13	114.58 (17)
C9—N1—Co1^i^	126.97 (14)	C11—C10—C13	123.95 (18)
C10—N1—Co1^i^	113.75 (13)	C10—C11—C12	118.96 (19)
C14—N2—C16	105.69 (17)	C10—C11—H11	120.5
C14—N2—Co1	127.21 (15)	C12—C11—H11	120.5
C16—N2—Co1	127.09 (14)	C11—C12—C8	120.62 (19)
C14—N3—C15	106.92 (17)	C11—C12—H12	119.7
C14—N3—C18	124.58 (18)	C8—C12—H12	119.7
C15—N3—C18	128.39 (17)	O3—C13—O4	125.53 (19)
C1—O2—Co1	151.15 (14)	O3—C13—C10	118.33 (17)
C13—O4—Co1^i^	117.89 (13)	O4—C13—C10	116.14 (17)
Co1—O5—H5A	124 (2)	N2—C14—N3	111.75 (19)
Co1—O5—H5B	129 (2)	N2—C14—H14	124.1
H5A—O5—H5B	106 (3)	N3—C14—H14	124.1
Co1—O6—H6A	122 (2)	C16—C15—N3	105.86 (19)
Co1—O6—H6B	123 (2)	C16—C15—H15	127.1
H6A—O6—H6B	109 (3)	N3—C15—H15	127.1
O2—C1—O1	126.48 (19)	C15—C16—N2	109.77 (19)
O2—C1—C2	116.35 (18)	C15—C16—H16	125.1
O1—C1—C2	117.17 (17)	N2—C16—H16	125.1
C3—C2—C7	118.53 (18)	C19^ii^—C17—C18	120.0 (2)
C3—C2—C1	120.86 (18)	C19^ii^—C17—H17	120.0
C7—C2—C1	120.59 (18)	C18—C17—H17	120.0
C2—C3—C4	120.86 (19)	C19—C18—C17	120.91 (19)
C2—C3—H3	119.6	C19—C18—N3	120.09 (19)
C4—C3—H3	119.6	C17—C18—N3	118.99 (19)
C3—C4—C5	120.90 (19)	C18—C19—C17^ii^	119.1 (2)
C3—C4—H4	119.6	C18—C19—H19	120.4
C5—C4—H4	119.6	C17^ii^—C19—H19	120.4
C6—C5—C4	117.91 (19)		
			
O5—Co1—N2—C14	76.7 (2)	Co1^i^—N1—C9—C8	172.91 (15)
O6—Co1—N2—C14	−100.1 (2)	C12—C8—C9—N1	−0.4 (3)
O4^i^—Co1—N2—C14	169.2 (2)	C5—C8—C9—N1	−179.25 (19)
N1^i^—Co1—N2—C14	170.6 (3)	C9—N1—C10—C11	2.4 (3)
O2—Co1—N2—C14	−13.3 (2)	Co1^i^—N1—C10—C11	−172.91 (17)
O5—Co1—N2—C16	−103.7 (2)	C9—N1—C10—C13	−176.73 (18)
O6—Co1—N2—C16	79.4 (2)	Co1^i^—N1—C10—C13	8.0 (2)
O4^i^—Co1—N2—C16	−11.3 (2)	N1—C10—C11—C12	−1.0 (3)
N1^i^—Co1—N2—C16	−9.9 (5)	C13—C10—C11—C12	178.0 (2)
O2—Co1—N2—C16	166.21 (19)	C10—C11—C12—C8	−1.2 (4)
O5—Co1—O2—C1	−82.1 (3)	C9—C8—C12—C11	1.9 (3)
N2—Co1—O2—C1	12.6 (3)	C5—C8—C12—C11	−179.4 (2)
O6—Co1—O2—C1	104.7 (3)	Co1^i^—O4—C13—O3	177.72 (17)
O4^i^—Co1—O2—C1	178.5 (3)	Co1^i^—O4—C13—C10	−2.5 (2)
N1^i^—Co1—O2—C1	−168.2 (3)	N1—C10—C13—O3	175.91 (18)
Co1—O2—C1—O1	−7.1 (5)	C11—C10—C13—O3	−3.2 (3)
Co1—O2—C1—C2	172.7 (2)	N1—C10—C13—O4	−3.9 (3)
O2—C1—C2—C3	20.7 (3)	C11—C10—C13—O4	177.0 (2)
O1—C1—C2—C3	−159.4 (2)	C16—N2—C14—N3	0.0 (3)
O2—C1—C2—C7	−157.4 (2)	Co1—N2—C14—N3	179.63 (15)
O1—C1—C2—C7	22.4 (3)	C15—N3—C14—N2	0.4 (3)
C7—C2—C3—C4	0.1 (3)	C18—N3—C14—N2	−176.1 (2)
C1—C2—C3—C4	−178.12 (19)	C14—N3—C15—C16	−0.7 (3)
C2—C3—C4—C5	2.3 (3)	C18—N3—C15—C16	175.6 (2)
C3—C4—C5—C6	−2.2 (3)	N3—C15—C16—N2	0.7 (3)
C3—C4—C5—C8	177.2 (2)	C14—N2—C16—C15	−0.5 (3)
C4—C5—C6—C7	−0.3 (3)	Co1—N2—C16—C15	179.93 (16)
C8—C5—C6—C7	−179.7 (2)	C19^ii^—C17—C18—C19	0.5 (4)
C5—C6—C7—C2	2.7 (3)	C19^ii^—C17—C18—N3	−179.3 (2)
C3—C2—C7—C6	−2.5 (3)	C14—N3—C18—C19	−143.7 (2)
C1—C2—C7—C6	175.7 (2)	C15—N3—C18—C19	40.6 (4)
C6—C5—C8—C12	−19.3 (3)	C14—N3—C18—C17	36.1 (3)
C4—C5—C8—C12	161.3 (2)	C15—N3—C18—C17	−139.6 (3)
C6—C5—C8—C9	159.4 (2)	C17—C18—C19—C17^ii^	−0.5 (4)
C4—C5—C8—C9	−19.9 (3)	N3—C18—C19—C17^ii^	179.3 (2)
C10—N1—C9—C8	−1.7 (3)		

Symmetry codes: (i) −*x*, −*y*+1, −*z*+1; (ii) −*x*+1, −*y*+2, −*z*.

### Hydrogen-bond geometry (Å, º)

**Table:**

*D*—H···*A*	*D*—H	H···*A*	*D*···*A*	*D*—H···*A*
O5—H5*A*···O3^iii^	0.81 (2)	1.89 (2)	2.697 (3)	178 (4)
O5—H5*B*···O1^iv^	0.82 (2)	2.01 (2)	2.825 (4)	175 (3)
O6—H6*A*···O3^v^	0.81 (3)	1.98 (3)	2.769 (3)	165 (3)
O6—H6*B*···O1^vi^	0.81 (2)	1.99 (2)	2.795 (3)	171 (3)

Symmetry codes: (iii) −*x*, −*y*+2, −*z*+1; (iv) *x*−1, *y*, *z*; (v) −*x*+1, −*y*+1, −*z*+1; (vi) *x*, *y*−1, *z*.
