# Supplementary figures and images for: Crystal structure of catena-poly[[di­aqua­cobalt(II)]-bis­[μ-5-(4-carb­oxy­ylato­phenyl)picolinato]-κ3 N,O 2:O 5;κ3 O 5:N,O 2-[di­aqua­cobalt(II)]-μ-1-[4-(1H-imidazol-1-yl)phen­yl]-1H-imidazole-κ2 N 3:N 3′]
Source: Acta Crystallogr E Crystallogr Commun. 2015 Jun 30;71(Pt 7):m145–6. doi: 10.1107/S2056989015012190 (PMC4518948; doi:10.1107/S2056989015012190)

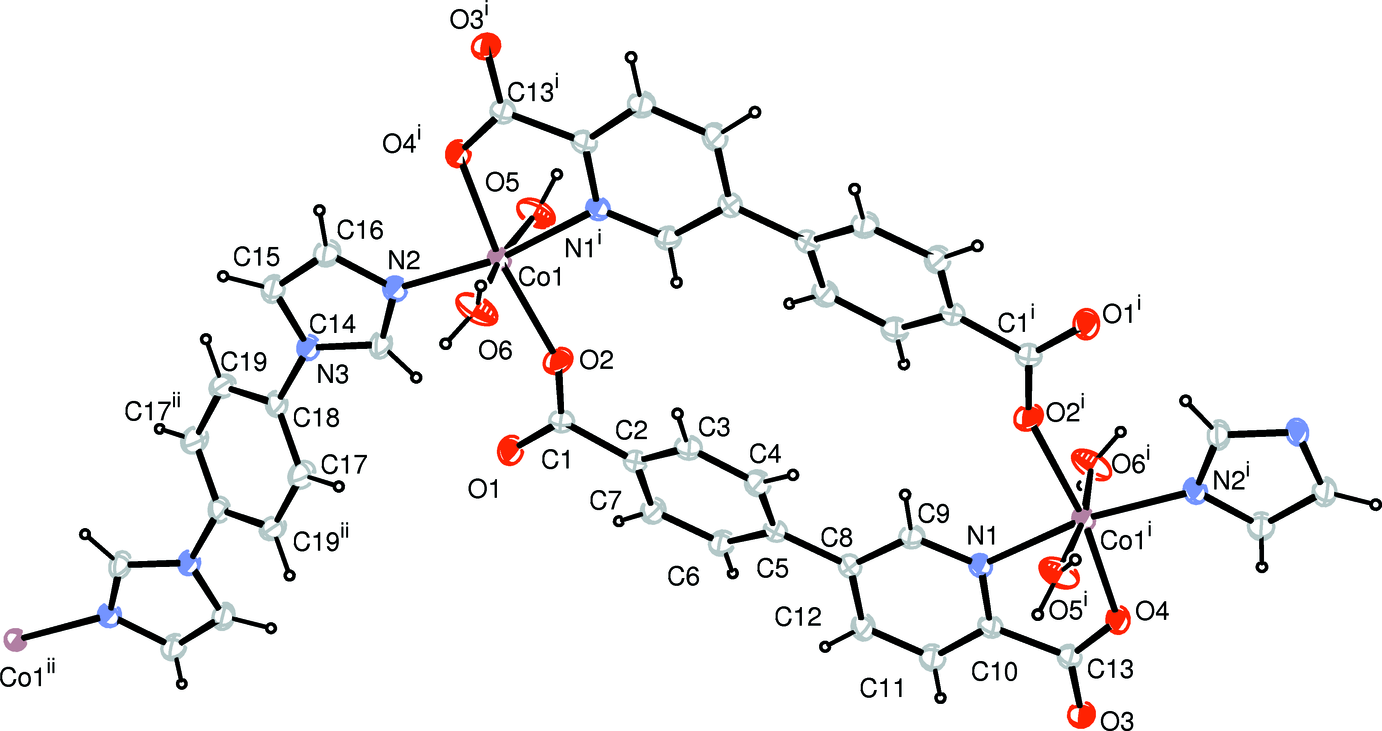

Supplement: Supplementary file 3 [file e-71-0m145-fig1.tif]
